# Spatiotemporal Dynamic Simulation of Acute Perfusion/Diffusion Ischemic Stroke Lesions Evolution: A Pilot Study Derived from Longitudinal MR Patient Data

**DOI:** 10.1155/2013/283593

**Published:** 2013-06-18

**Authors:** Islem Rekik, Stéphanie Allassonnière, Stanley Durrleman, Trevor Carpenter, Joanna Wardlaw

**Affiliations:** ^1^Division of Neuroimaging Sciences, Brain Research Imaging Centre (BRIC), Edinburgh University, UK; ^2^CMAP, Ecole Polytechnique, route de Saclay, 91128 Palaiseau, France; ^3^INRIA, ICM Pitié-Salpétrière Hospital, Paris, France

## Abstract

The spatiotemporal evolution of stroke lesions, from acute injury to final tissue damage, is complex. Diffusion-weighted (DWI) and perfusion-weighted (PWI) imaging is commonly used to detect early ischemic changes and attempts to distinguish between permanently damaged and salvageable tissues. To date, 2D and 3D measures of diffusion/perfusion regions at individual timepoints have been widely used but may underestimate the true lesion spatio-temporal dynamics. Currently there is no spatio-temporal 4D dynamic model that simulates the continuous evolution of ischemic stroke from MR images. We determined whether a 4D current-based diffeomorphic model, developed in the field of statistical modeling for measuring the variability of anatomical surfaces, could estimate patient-specific spatio-temporal continuous evolution for MR PWI (measured as mean transit time, (MTT)) and DWI lesions. In our representative pilot sample, the model fitted the data well. Our dynamic analysis of lesion evolution showed different patterns; for example, some DWI/PWI dynamic changes corresponded with DWI lesion expansion into PWI lesions, but other patterns were much more complex and diverse. There was wide variation in the time when the final tissue damage was reached after stroke for DWI and MTT.

## 1. Introduction

Stroke is the third leading cause of death in industrialized countries [1], the second commonest cause of death worldwide [2], and a major social and financial burden. 80% of stroke is ischemic, and so far the only effective treatment is with thrombolytic drugs to dissolve the occluding thrombus. Despite the considerable potential benefit, these treatments are potentially hazardous; for example, rt-PA increases the risk of haemorrhagic transformation to around 5% [3]. Many have suggested that their use could be focused on patients who are most likely to benefit by using techniques such as Magnetic Resonance (MR) imaging to identify the location and spatial extent of ischemic tissue and differentiate salvageable from nonsalvageable tissues [4, 5]. MR perfusion and diffusion imaging (PWI, DWI, resp.) are thus increasingly used in clinical trials and treatment planning, but it is as yet unclear how best they should be used to guide treatment decisions [6].

**Figure 1:**
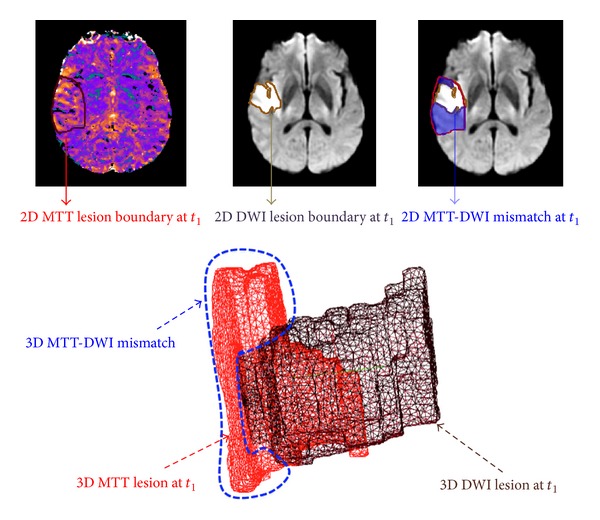
MTT-DWI mismatch at first acquisition acute timepoint *t*_1_. Top row: MTT-DWI mismatch area in blue visualised on 2D MR axial slices. Bottom row: the 3D reconstruction of MTT and DWI surfaces and their corresponding mismatch spatially defined as the “output space” produced when taking the diffusion volume out of the perfusion one, outlined in blue.

Numerous medical image analysis approaches have been applied to DWI and/or PWI data using 2D or 3D approaches to determine how the ischemic stroke lesions evolve and what factors influence this. Most of these studies evaluated MR (or CT) images of ischemic stroke as static “snapshots” to predict tissue prognosis and its association with clinical outcome [5, 7]. These studies suggest considerable potential to identify the change in DWI hyperintense and PWI hypoperfused tissues using pixel-by-pixel algorithms or volumetric region of interest analyses [8–13]. Although many studies to date suggest that some patients conform to the expected growth of the DWI into the PWI lesion [14–16], others did not. This might be due to insensitivity of visually assessed 2D lesions [17], and voxel-based analyses may be more sensitive [6, 10, 18]. Many studies have tested different DWI or PWI thresholds to define their respective lesions, but there is still considerable variation in final tissue outcome that is not completely explained by the DWI/PWI mismatch hypothesis [19–22]. A larger perfusion deficit surrounding the visible diffusion lesion—commonly referred to as the “mismatch”—is thought to contain “at risk” or “penumbral” tissue, and the DWI lesion is commonly considered to be non-salvageable. The perfusion-diffusion mismatch can also be viewed as a 3D spatial discrepancy (or a volumetric difference) between the PWI brain volume and DWI lesion volume or some portion therein [6, 22] (Figure 1). The mismatch hypothesis has been tested in randomized clinical trials and observational studies such as the Echoplaner Thrombolysis Evaluation (EPITHET) trial and the DWI Evolution for Understanding Stroke Etiology (DEFUSE) study. Although patients with MR mismatch are thought to be more likely to respond to treatment, other evidence indicates that even if reperfusion occurs quickly, the outcome may not conform to the expected evolution pattern [23, 24].

One explanation for this variation in behavior in DWI and PWI lesions is that methods examining large regions of interest or even voxel-by-voxel-based analyses may not adequately capture the dynamic changes occurring between individual timepoints and their relationship to anatomically important brain regions. A mathematically well-defined 4D dynamic model may help overcome these limitations by providing more detailed and anatomically relevant information on stroke evolution. There is currently no 4D dynamic model available that simulates the continuous evolution of ischemic stroke based on MR or other image types [25]. In the present paper, we present the proof-of-concept application of a novel spatiotemporal model to DWI/PWI lesions that has not previously been applied in stroke. We recognize that multiple patient-dependent, stroke-dependent and imaging-dependent factors govern the dynamic evolution of ischemic brain injury and its detection [25], and therefore that it may be very difficult to find one PWI or DWI threshold or any one imaging characteristic that fits an entire population of stroke patients [19, 20]. Furthermore, perfusion lesions can be visualized using different perfusion parameter maps, for example, cerebral blood flow (CBF), cerebral blood volume (CBV), mean transit time (MTT), and so forth [5, 24]; for simplicity at this stage of “proof-of-concept” in model development, we used the MTT lesion.

To capture the individual differences and enable a model that is as patient specific as possible, we chose a 4D spatiotemporal approach that captures individual differences independent of clinical factors such as stroke severity, the effected vascular territory, or the location of the occluded artery and that did not require any information except for the DWI and PWI lesion outlines. The model is described in detail in the methods section. We used the model to address three aspects of stroke lesion evolution where imaging could provide valuable insight.

Firstly, we modeled individual DWI and PWI lesion dynamic changes separately to identify subregional differences in expansion and contraction of the respective DWI and PWI lesions and determined how well the model fitted the true lesion evolution as represented by the original image data.

Secondly, we explored the pattern of change in the DWI and PWI lesions in relation to each other in individual patients, imputed to 3 hourly time intervals, from the three acquired imaging snapshots spaced many days apart.

Thirdly, we investigated the models prognostic potential by identifying the timepoint at which the PWI/DWI lesions best matched the final observed T2 lesion as an indicative estimate of the “final” lesion damage.

Finally, we would like to emphasize that the aims of the present work were to (a) demonstrate that 4D modeling was possible in stroke, (b) identify the limitations to this approach and potential solutions, and (c) demonstrate the considerable potential for the 4D modeling in advancing our understanding of the dynamics of ischemic stroke lesion evolution.

## 2. Material and Methods

### 2.1. Data Selection and MRI Acquisition

#### 2.1.1. Data Selection

In order to apply and test this model, we selected eight representative patients (5 males, 3 females) from a study of MR imaging in hyperacute stroke of original sample size 48. Primary study methods were previously described [26]. These 8 patients represented a range of stroke severity (NIHSS = 11.63 ± 7.8), age (72 ± 5.2 years), and all patients had solitary acute ischemic lesions on DWI and PWI with evidence of mismatch (i.e., MTT > DWI) on MTT. Additionally, for the initial application of the model we required that (i) the MTT and DWI lesions should both be visible at, at least, 3 timepoints, (ii) the MTT/DWI mismatch should be larger than 3 cm^3^, and (iii) the lesion consisted of one solitary lesion only and this did not vary between timepoints (i.e., we avoided lesions consisting of several separate acute lesions or where the number of lesions varied between timepoints). We chose this limit to minimize resolution artifact problems during this proof-of-concept phase of model application. To further simplify the model development, we excluded patients who received thrombolytic or other reperfusion therapies. All model development and analyses were performed blind to all clinical data. The first acquisition timepoint was at around 5 hours, the second at around 5 ± 1 days, and the third at 10.5 ± 2.5 days after stroke [26]. The median DWI lesion volume was 43.42 cm^3^ and MTT volume was 66 cm^3^ when measured by manual outlining in the original study from which the acute data were taken.

#### 2.1.2. MRI Acquisition and Preprocessing Steps

 All MR images were acquired using a GE Signa LX 1.5-T MR scanner (General Electric, Milwaukee, WI, USA) with a birdcage quadrature coil and a standardised protocol for acute stroke [26]. The spin-echo echoplanar imaging diffusion tensor axial sequences and dynamic susceptibility contrast echoplanar imaging PWI had 15 axial slices each of 6mm thickness with an interslice gap of 0.97 mm and an imaging matrix 128 × 128 encompassing a 240 × 240 mm field of view. MTT perfusion maps were generated using PWI data, and full details of the image acquisition and processing protocol have been described previously [26]. The DWI, MTT, and final follow-up T2-weighted images (at ≥1 month after stroke) were coregistered, and their corresponding lesions manually were delineated on every slice on which they were visible by an expert. For the model we preprocessed the images to subsample slice thickness and reduced it to 1 mm by splitting each 6 mm voxel into 6 subvoxels along the *z*-axis. We used unthresholded MTT at this stage in model application as it generally provides a single contiguous lesion.

### 2.2. Description of the Model: A Current-Based Longitudinal Shape Deformation Model

A wide range of mathematical studies have focused on computing differences between consecutive scans of the same subject or objects and extracting key features to compare them, for example, to a population. Recently, Durrleman et al. [27–29] introduced a more generic approach to investigate geometric variability of anatomical structures. Durrleman's approach has the key features of two previous mathematical approaches: Kendall's approach [30] which focused on only capturing geometrical variations and used a metric defined in the space of shapes and measuring shape change, and Grenander's approach [31] which added the “texture part,” also known as the “deformation residual,” and used a metric defined in the space of deformation. In merging both approaches, the distance between different shapes becomes a measure quantifying the “optimal” deformation for morphing one shape onto another. A further step was to use the framework of currents, introduced by Vaillant and Glaunès in [32], to define the deformation metric and the derived distance between shapes, as this was important for dealing with arbitrary nonuniform anatomical shapes.

This key step improved the generic ability of the new model [28] to deal with any kind of data: for example, surface shapes such as the skulls of bonobos and chimpanzees [29] and curves such as white matter bundles [27, 28]. The major achievement of this approach is that it is subject specific and that

- this mathematical representation of surfaces does not require all objects to have the same number of landmarks (normals) to compute the distance between surfaces;
- the metric of currents densely conserves all the geometric properties of the data (here the normal to the surface) up to a given scale.

Furthermore, Durrleman's approach allows vector features (momentum and velocities of deformation) to be extracted and compared between surfaces. Unlike approaches based on extracting scalar features such as rate of growth, these vector quantities allow more informative mathematical features—such as local curvature and orientation of the shape surface—to be used.

The name “currents” comes from the introduction of a more mathematically precise “way of perceiving a surface,” inspired from Faraday's law of induction in physics, where the variation of a magnetic vector field *B* through a surface *S* induces a current within a wire loop *S* delimiting *S*. The intensity of the current is proportional to the variation of the flux of this magnetic field Φ(*B*) = ∫_*S*_ *B* ^*T*^ *ndλ*, with *dλ* Lebesgue measure on the surface. Therefore, as we measure the intensity of the current within the wire *via* the flux Φ(*B*) for every possible spatial variation of the magnetic field, the quantitative relationship allows us to retrieve the geometry of the wire. Hence, as a current object, a 3D meshed surface *S* can morphometrically be fully characterized by a collective measure of fluxes associated with every possible vector field *ω* defined in a space *W* and encoded by the 3D surface unit normal vectors *n* localized at the mesh cell center *x* of each one of the objects facets.

In our case, the space that contains “the magnetic vector field” traversing the shape surface *S* is called a “test space *W*” and is defined as a “reproducing kernel Hilbert space (RKHS)” with a regularity parameter *λ*_*W*_ tuning the regularity scale at which local curvature of the surface is captured or overlooked (e.g., curvatures with radius <*λ*_*W*_ are “visually” unnoticed from the current-based shape modeling perspective).

This sets out a more realistic and efficient framework to represent stroke lesion surfaces as dynamic (rather than static) structures which contract, expand, swell, and shrink in different directions, with variation in the speed of these local changes, without imposing prior assumptions. To estimate the pattern of change in ischemia, we used a sequence of time-indexed surfaces {*S*_0_, *S*_1_,…, *S*_*n*_} from timepoints *t*_0_ to *t*_*n*_.

Considering a source baseline shape *S*_0_ (in our case the 3D meshed lesion surface at the first acquisition timepoint) and {*x*_*p*_}_{1,…,*N*}_ set of points representing the centers of facets of *S*_0_, the ultimate goal of the model is to estimate a continuous evolution deformation function *χ*(*t*) deforming the source shape *S*_0_ successively onto the next shape until reaching the final shape *S*_*n*_: *S*(*t*) = *χ*_*t*_(*S*_0_). A diffeomorphic flow equation (*dχ*_*t*_(*x*)/*dt* = *v*_*t*_(*χ*_*t*_(*x*)), *t* ∈ [0, *T*], with *χ*_0_ = Id), guided the deformation function *χ*_DWI_^*v*^ (resp., *χ*_MTT_^*v*^) of the DWI (resp., MTT) lesion shapes, using a dense and smooth kernel-induced vector field, without assuming any prior anatomical spatial constraints or any interdependencies of spatiotemporal MTT/DWI shapes. Note that the model does not allow lesion behavior such as folding, tearing, or shearing, but, in the case of stroke, we do not expect these behaviors anyway.

To ensure that the solution at the final time step (*t* = 1) of this flow equation is a piecewise geodesic diffeomorphism that best matches the set of our time-indexed currents (surfaces), the time-varying speed vector field (*v*_*t*_)_*t*∈[0,1]_ must belong to a space of smooth vector fields *V* [33]. The smooth deformation vector space *V* belongs to an RKHS with a Gaussian kernel *K* ^*V*^ and a standard deviation parameter *λ*_*V*_ determining the scale under which deformations are locally similar to the identity map (no deformation). Thus, at a specific timepoint *t*, the velocity of the evolution of the surface mesh, with *x* as its center, is defined as a sum of convolutions between the Gaussian deformation kernels, respectively, located at all centers *x*_*p*_ of the surface meshes, and their corresponding momenta *α*_*p*_(*t*) guiding the deformation trajectory: *v*_*t*_(*x*) = ∑_*k*=1_^*N*^ *k*_*V*_(*x*_*k*_(*t*), *x*)*α*_*k*_(*t*).

The integration of the estimated velocity field, ultimately, defines the deformation trajectories as geodesic paths between diffeomorphisms. To ascertain these smooth trajectories morphing one shape into another passing by the observed timepoints, we introduce a temporal regression functional *J*(*v*) that we minimize through running a gradient descent algorithm as described in [28] (see Appendix). The minimum of the functional *J*(*v*) achieves simultaneously (a) the minimum of the regularity of the deformation that represents physically the kinetic energy of the estimated deformation and (b) a second term that represents the fidelity of the estimated data to the observed data.

The estimation of the functional *J*(*v*) depends on four main parameters, which are automatically fixed as follows: a scalar parameter *γ* representing a trade-off between the regularity and the fidelity to data terms set to 10^−4^, a scalar parameter denoting the deformation scale *λ*_*V*_ above which points move in an uncorrelated way set to 30% of the bounding box enclosing the three observed lesions, a scalar parameter *λ*_*W*_ characterizing the regularity current-related scale under which the algorithm will be blind to shape regularities variations set to 5 mm or 35 mm depending on the observed shape regularity, and a time discretization step *δt* set to 3 hours.

The ultimate estimation of the “spatiotemporal” variability in DWI and MTT lesions will provide us with a clear representation of the similarities and differences between MTT and DWI kinetic patterns and thereafter to evaluate some biological assumptions.

### 2.3. Methodological Tools for Comparing the Estimated PWI and DWI Lesion Evolution

**Figure 2:**
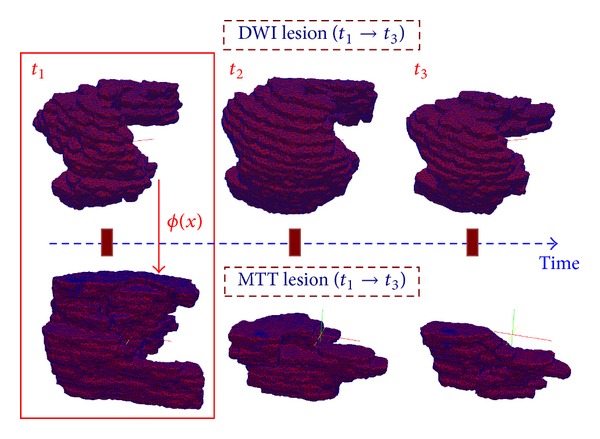
3D meshed reconstruction of DWI and MTT lesions at three acquisition timepoints. An additional spatial deformation *ϕ* mapping DWI into MTT at *t*_1_ is estimated, also noted as *ϕ*_*t*_1__.

In order to assess the dynamic changes in PWI and DWI lesions across three successive timepoints from acute and subacute stages (from *t*_1_ to *t*_3_), we used the manually delineated PWI and DWI lesions and reconstructed their surfaces as time-indexed sets of shapes embedded in a three-dimensional space (Figure 2). Observing DWI/MTT lesions in three dimensions provides us with more specific spatial information about their shapes, in terms of the geometric mathematical constructs of “cavities,” “curvatures,” “compactness” and “closeness.” The analysis of the baseline surface (*S*_0_) deformation with time provides an estimate of the diffeomorphic dynamic patterns of change that we will refer to as the estimated evolution scenario and that fully describes the kinetic change driving the contractions and expansions of different areas of the lesion surface from the initial timepoint.

To show the differences and similarities in the dynamic behavior of the MTT and DWI lesions as they evolve over time, we needed to define appropriate mathematical tools that would adequately set a connection between the changes in the DWI lesion and the MTT lesion as they both evolved with time. This connection is defined as a geodesic diffeomorphism (smooth deformation with a smooth inverse) *ϕ*_*t*_(*x*), that automatically identified corresponding areas in MTT and DWI baseline surfaces. We used the same framework of currents as above to estimate this additional deformation function. Thus we estimated this additional spatial deformation *ϕ*_*t*_1__(*x*) by mapping each DWI lesion surface mesh onto the MTT lesion surface mesh at *t*_1_, thereby avoiding any inter- and intraobserver variability that would inevitably result from any attempt to match these anatomical points manually. Spatially connecting the DWI and MTT lesions through identifying the correspondences between them was achieved through the same energy minimization scheme between the two baseline shapes *S*_DWI_(*t*_1_) and *S*_MTT_(*t*_1_), as introduced earlier in the previous section.

**Figure 3:**
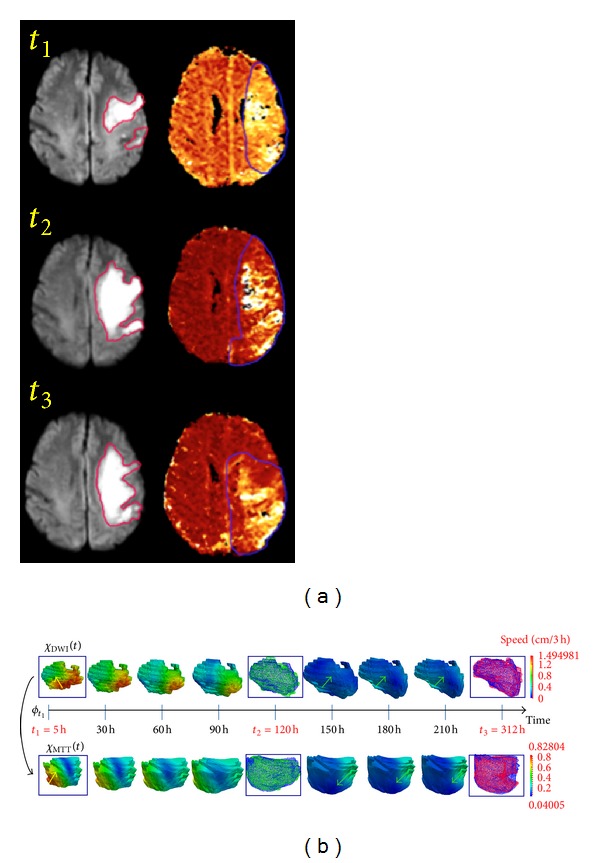
Estimated DWI/MTT lesion evolution scenarios. (a) DWI (versus MTT) axial slice at 3 timepoints in the first (versus second) column. (b) Estimated DWI/MTT evolution scenarios with estimated velocity norm (speed) in cm/3h. In the Supplementary Material of this paper, we have included a video (Video 1) which visualizes the 4D evolution of both MTT (in red) and DWI (in blue) lesions—as if in “real” time. At *t*_2_ (versus *t*_3_), the green (versus red) surface represents the observed lesion, and the blue one represents the estimated lesion. Green arrows represent contraction areas and yellow ones expansion areas.

Note that the estimation of *ϕ*_*t*_1__(*x*) is not constrained by any anatomical paths and does not require any initially selected anatomical landmarks. We used the new estimated final shape ${\tilde{S}}_{\text{MTT}}(t_{1})=\phi_{t_{1}}(S_{\text{DWI}}(t_{1}))$ as the baseline lesion shape for the MTT scenario at *t*_1_ to estimate the perfusion lesion evolution function *χ*_MTT_^*v*^. Therefore, we can now spatially link both estimated MTT and DWI evolution scenarios, respectively, noted as *χ*_MTT_^*v*^ and *χ*_DWI_^*v*^, by using *ϕ*_*t*_1__ and its inverse *ϕ*_*t*_1__^−1^ as a continuous junction to connect DWI and MTT lesions at the first timepoint (Figures 2 and 3). We can also follow back in time the spatiotemporal change respectively, within each MTT and DWI lesion evolution pattern by using the inverses of the estimated evolution functions *χ*_MTT_^−1^(*t*) and *χ*_DWI_^−1^(*t*). Knowing that we can navigate throughout each DWI and MTT 4D evolution scenario separately, we can extract the kinetic similarities and differences in anatomically corresponding DWI versus MTT lesion evolution as follows.

- We firstly compute the norm of the speed at each time step *t*_*i*_ at each point of the evolving lesion 3D surface.
- We then extract contracting (corresponding to inward speed direction to the surface) and expanding (corresponding to outward speed direction to the surface) local areas at *t*_1_ of the DWI-estimated evolution scenario *χ*_DWI_^*v*^(*t*). We compute the mean evolution speed of the contraction (versus expansion) areas at each time step of *χ*_DWI_^*v*^(*t*). Using the mean evolution speed minus (versus plus) its standard deviation as an automatic contraction (versus expansion) threshold, we only mark areas with high contractions (versus expansions) from the DWI lesion surface at *t*_1_ and then spatially map them into the MTT lesion at *t*_1_, as deformed by *ϕ*_*t*_1__.
- Finally we compute the mean speed for these contracting and expanding areas over their spatiotemporal evolution for the 8 representative patients, in both DWI and MTT abnormalities.

### 2.4. Identification of the Time at Which the DWI/MTT Lesions Matched the Final T2 Lesion

**Figure 4:**
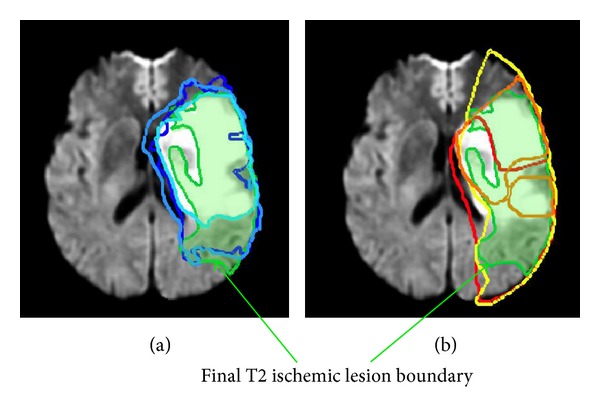
Time-indexed penumbra and core lesion boundaries combined to final T2-weighted boundary imaged at >1 month. (b) MTT manually delineated penumbra boundaries consecutively at *t*_1_ (in orange), *t*_2_ (in yellow), and *t*_3_ (in red). (a) DWI lesion boundaries ranging from light to dark blue as time evolves from *t*_1_ to *t*_2_ and then *t*_3_. In both MR axial slices, the final T2 boundary is displayed in green shading.

The ability to know precisely at which time the DWI and MTT lesions are most spatially coherent with the final T2-weighted lesion means that we can better evaluate the prognostic potentials of DWI and MTT modalities. To our knowledge, up to this date, detailed information on 4D lesion shape change between one acquisition timepoint and the next is limited [25]. For instance, as shown in Figure 4, the details of the mechanism of the penumbra boundary at *t*_1_ (in orange) extending into the yellow manual outline at *t*_2_ and then shrinking back to the red boundary at *t*_3_ while intercepting the green T2-weighted boundary remains unclear and quite difficult to accurately envision in space and time. We determined the exact timepoints *t*_DWI,T2_ and *t*_MTT,T2_ (in hrs from stroke) where the estimated DWI/MTT lesion boundaries were spatially the closest to the final T2 lesion surface *S*_T2_ by computing the dice index—a measure that quantifies the volumetric overlap between the estimated lesion volume delimited by the dynamic surface and the final static T2 lesion volume and ranges from 0 to 1—at each time step *t*_*i*_ as follows:

(1) diceDWI(t)  =2V(χDWIv(SDWI,t1))∩V(ST2)V(χDWIv(SDWI,t1))+V(ST2), t∈[t1,t3].

We also computed, at each time step *t*_*i*_, the symmetric distance—averaging the symmetric closeness of both shapes in millimeters—to provide a quantitative spatial discrepancy measure between the time-varying DWI and MTT surfaces (*S*_DWI_(*t*) and *S*_MTT_(*t*)) and the static final T2 lesion *S*_T2_ and establish a more intuitive understanding of how close the dynamic surfaces get to the static T2-weighted surface. Noting that *χ*_DWI_^*v*^(*S*_DWI,*t*_1__) = *S*_DWI_(*t*), we have

(2) dsym(t)=1|ST2|+|SDWI(t)|×[∑x∈ST2min⁡⁡ d(x,SDWI(t))  +∑x∈SDWI(t)min⁡⁡ d(x,ST2)],

where *d* denotes the closest Euclidean distance between a point and a surface.

The last step was to identify the timepoint *t*_DWI,T2_^dice^ (resp., *t*_DWI,T2_^*d*_sym_^), where *S*_T2_ is closest to *S*_DWI_(*t*) by seeking the maximum over *t* ∈ [*t*_1_, *t*_3_] of dice_DWI_(*t*) (resp., the minimum of *d*_sym_(*t*)):

(3) $$\begin{array}{c} t_{\text{DWI},\text{T}2}^{\text{dice}}=\underset{t\in [t_{1},t_{3}]}{\max}\text{dic}{\text{e}}_{\text{DWI}}\left(t\right). \end{array}$$

In a similar way, we computed the same measures for the MTT estimated lesion evolution. These specific identified timepoints (*t*_dice_ and *t*_*d*_sym__) would, for example, give us insights into the hypothesis that acute DWI abnormality is a surrogate marker for permanently dead tissue, as well as the hypothesis that acute MTT surface boundary is the maximum boundary of infarct progression.

## 3. Results

### 3.1. Evaluation of the Estimated MTT and DWI Spatiotemporal Lesion Evolution

We evaluated the accuracy of our estimation of DWI and MTT lesion evolution by computing the mean and standard deviation (SD) values of the dice index between the estimated and the true lesion volumes at the second and third timepoints for the 8 patients. We found good agreement between the estimated lesion evolution scenarios for MTT and DWI lesions and the observed samples (mean dice SD values: MTT (*t*_2_: 0.76 ± 0.09; *t*_3_: 0.8 ± 0.08) and DWI (*t*_2_, *t*_3_: 0.9 ± 0.02)) (Table 1). Figure 3 shows an example of how the model visualizes the kinetic change in DWI and MTT lesion shapes. The color of the dynamic surface indicates the estimated speed at which the lesion surface is contracting or expanding: changes from blue to green, and then yellow to red indicate increasing speed of change. This figure shows representative snapshots at equal time intervals across the entire estimated lesion evolution scenario. A video of the entire 4D simulation of MTT (in red) and DWI (in blue) lesion (see Supplementary Material available online http://dx.doi.org/10.1155/2013/283593, Video 1), provided in Supplementary Material, is much more informative. Additionally, we can also see clearly the very good agreement between the model's estimated time-evolving blue lesion surface and the original lesions at *t*_2_ (in green) and at *t*_3_ (in red).

### 3.2. Comparison of the MTT and DWI Kinetic Patterns

We compared the deformation of corresponding DWI and MTT lesion regions to determine any correlations between their contractions and expansions. We first examined lesion behaviour in individual patients and then identified common patterns of lesion change across all eight representative patients.

#### 3.2.1. Individual Observations

**Figure 5:**
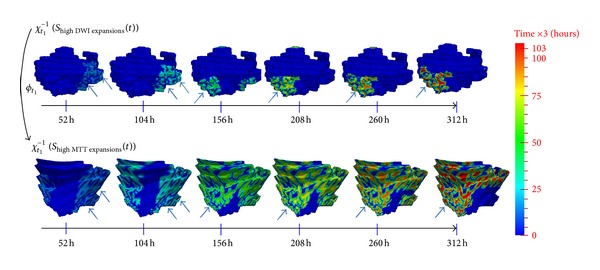
Comparison between highly expanding DWI and MTT areas extracted across the spatiotemporal estimated scenarios. As the color ranges from blue to green to red, the different successive areas with high expansion speed are “lit up” at each time step, all mapped back at the baseline lesion shape acquired at *t*_1_ using the inverse of the estimated evolution function *χ*_*t*_1__^−1^(*S*_highDWIexpansions_(*t*)) for DWI lesion and *χ*_t_1__^−1^(*S*_highMTTexpansions_(*t*)) for MTT lesion. The mapping back enables us to foresee the upcoming dynamic changes—more precisely expansions—on the acute baseline lesion surface. In the Supplementary Material of this manuscript, we included Video 2 (resp., Video 3) where you can see the “lighting up” process of successively highly expanding DWI (resp., MTT) regions along the evolution process; all are visualized on the baseline surface at the first acquisition timepoint *t*_1_. The blue arrows point to highly expanding areas in DWI lesion and their corresponding areas that also expanded in the MTT lesion. The dark blue areas—that did not change color as time evolved—did not expand; that is, they contacted.

**Figure 6:**
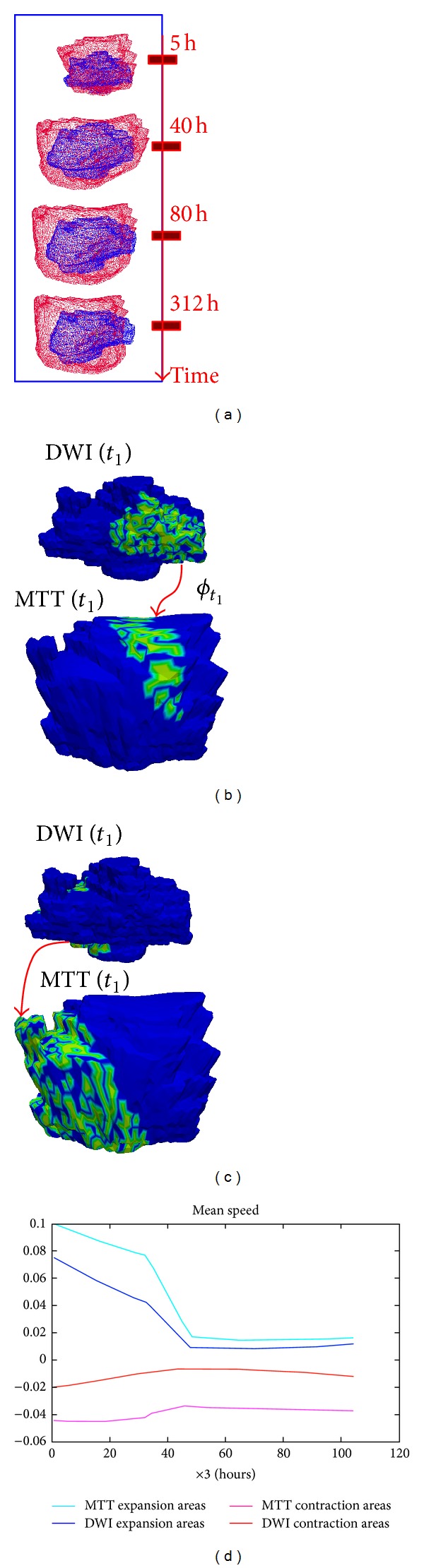
Patient-specific estimated kinetic patterns of change in one patient. (a) The estimated spatiotemporal evolution of DWI (in blue) and MTT (in red) lesions. (b) Detection of highly contracting (versus expanding in (c)) areas in DWI lesion at *t*_1_ and spatially mapping them into MTT lesion at *t*_1_. (d) Spatiotemporal mean speed evolution of highly contracting and expanding DWI lesion areas for this patient and their corresponding areas in MTT lesion.

Figure 5 shows in a single subject of the spatial distribution of highly expanding areas on the DWI lesion surface and their corresponding areas on the MTT lesion surface using the additional deformation *ϕ*_*t*_1__. The change of color from blue to green and to red indicates the progress in time from *t*_1_ to *t*_3_. We also used the inverse of the estimated DWI evolution function *χ*_*t*_1__^−1^(*S*_highDWIexpansions_(*t*)) to map back all highly expanding DWI areas at later timepoints onto the static baseline acute DWI surface. The DWI areas that expanded quickly from the acute surface are shown successively highlighting the “new” expansion areas at each subsequent timepoint (see Supplementary Material, Video 2). Similarly, we also highlighted consecutive areas of expansion on the baseline acute MTT lesion. This facilitates the comparison of areas of subsequent expansion on the static acute DWI and MTT lesions (Figure 5). In this one subject, there were areas where the DWI lesion expanded into the MTT lesion (blue arrows point out to spatially corresponding local areas that simultaneously expanded), but there were also areas where the opposite occurred; that is, DWI shrank (dark blue contractions) where MTT expanded (areas not marked by blue arrows) or DWI expanded into an area of MTT contraction. We found similarly varied patterns in other subjects; for example, Figure 6 shows limited correlation between high contracting (versus expanding) areas of the DWI surface and their corresponding areas in MTT surface at the acute stage. It also shows how MTT lesion surface (in red, Figure 6(a)) started by rapidly expanding (light blue curve in Figure 6(d)) then went through an ultimate phase of shrinking in the space where the DWI lesion (in blue) expanded.

#### 3.2.2. Population-Based Observations

**Figure 7:**
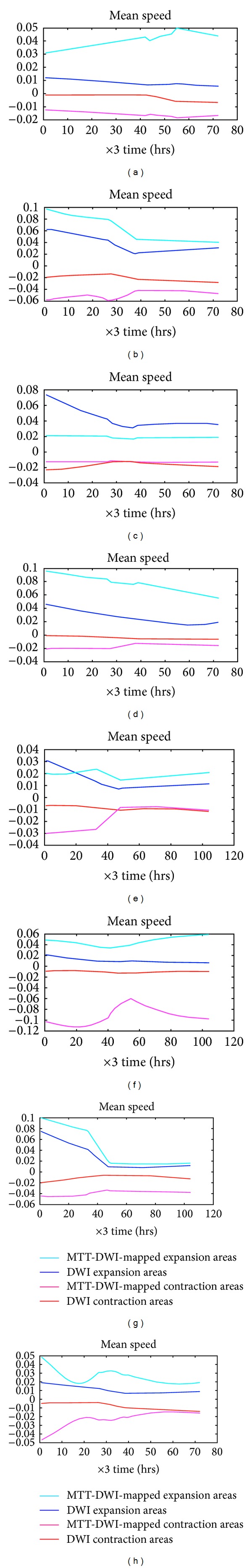
Spatiotemporal mean speed evolution of DWI high contraction and expansion lesion areas for the 8 representative patients and their corresponding areas in the moving MTT lesion. Each box represents a different patient.

We plotted the mean speed of highly contracting and expanding areas for the eight representative patients (Figure 7). This figure demonstrates that DWI areas with large contractions changed faster than their corresponding regions within the MTT lesion. In 6 patients, rapidly expanding DWI areas changed more slowly than their corresponding areas in the MTT lesion. In other words, expanding MTT areas and shrinking DWI areas displayed the speediest dynamic change. While some patients showed a monotonic (i.e., entirely increasing or decreasing) mean speed evolution, others showed both MTT and DWI contracting and expanding areas. The large variation in the increasing/decreasing mean speeds with fluctuations made it difficult to see any common monotonic kinetic evolution pattern in both MTT and DWI lesions between patients.

### 3.3. Localization in Space and Time of Final Imaged T2-Outcome in DWI/MTT Estimated Evolution Scenarios

We computed the two complementary measures (the dice index and the symmetric distance). We then extracted the corresponding timepoints (in hrs) where the maximum spatial concordance between the two shapes occurred. Both measures produced quite similar results as follows (Table 2).

- Maximum special closeness between DWI/MTT surface and T2 surface: the dice index averaged for the 8 patients for the spatiotemporal DWI and MTT lesion evolution versus final T2 lesion indicated wide inter-patient variation (DWI dice range 0.0008 to 0.77, median 0.58; MTT dice range 0.003 to 0.63, median 0.39) and similarly wide variation in the temporal symmetric distance for DWI 3.8 to 24.2, median 6.1 mm and MTT 6.1 to 33.2, median 9.8 mm versus final T2. In 5/8 patients, the mean DWI-T2 overlap volume over time exceeded 55% (corresponding to less than 6.2 mm in symmetric distance). This indicates that in most cases there is a good geometric concordance between the T2 and DWI surfaces although this did not necessarily occur at the subacute stage (last acquired DWI image) that is in disagreement with the assumption that acute DWI will grow into the final T2 lesion. Three patients showed almost no MTT-DWI spatiotemporal lesion evolution overlap versus final T2 (mean dice index <0.05). The fact that the dice index did not converge to 1 and the symmetric distance did not fall to 0 mm shows that the time-evolving DWI abnormality does not converge to the final T2 boundary but rather encounters it as it progresses or regresses with time.
- Time of maximum DWI/MTT surface overlaps with the final T2 surface: the time *t*_DWI,T2_^dice^ when DWI most closely matched final T2 lesion ranged from 6 to 237 hrs, median 138 hrs from stroke onset. For MTT the time of best match to final T2 *t*_MTT,T2_^dice^ ranged from 9 to 270 hrs, median 78 hrs. Five patients showed better later in time geometric concordance of DWI than MTT with final T2 boundaries. Furthermore, the time of appearance of the final T2 lesion according to the symmetric distance varied considerably between MTT and DWI lesion evolution scenarios in different patients. In four patients the estimated MTT 4D scenario met the final T2 lesion boundary earlier than did the DWI lesion (*t*_MTT,T2_^*d*_sym_^ > *t*_DWI,T2_^*d*_sym_^), partly supporting the assumption that the MTT lesion extent in the acute phase may indicate the final irreversibly damaged ischemic tissue better than the acute DWI lesion.

## 4. Discussion

We applied a 4D model to evaluate the change in acute stroke lesions from the first few hours to 1–3 months after stroke, to our knowledge, the first time a 4D model has been used to simulate the dynamic evolution of stroke lesions [25]. The current-based longitudinal shape regression model—based on an energy minimization problem derived from a diffeomorphic flow equation—provided a simulation of lesion surface evolution that fitted well to the true data. It also demonstrated the potential to obtain more insights into the spatiotemporal behaviour of acute stroke lesions within an anatomically specific space. While some features fitted the expected patterns of lesion growth into tissue at risk, other patterns did not. Although this study is too small to determine reliably any relationship between estimated contraction and expansion speeds and final outcome, it demonstrated the proof of principle that a 4D model can provide a solid basis for examining similarities and differences between DWI and MTT kinetic evolution patterns and thence provide a framework on which to investigate factors that influence infarct evolution. These might include the effect of leukoaraiosis adjacent to the DWI lesion, anatomic structures like sulci that constrain growth, or treatment factors (e.g., use of hypothermia or thrombolysis) that may affect these in a much wider range of patients.

This model has some innovative mathematical aspects that we took advantage of, such as (a) there is no need for a point-to-point landmark matching between consecutive surfaces since the metric of currents does not assume any *prior* landmark matching, (b) the metric of currents conserves all geometric properties of the surface such as curvature, and (c) embedding these 3D lesion shapes into an RKHS space, which is a dense span of vector fields and equipped with an inner product, provides a robust numerical framework to efficiently estimate morphological and kinetic changes in evolving shapes. Furthermore, the model allows an examination of the whole time course (from 3 hrs to the latest imaging time) dynamically as shown in the videos (Supplemental Material) or to obtain “snapshots” of directions and speeds of contraction and expansion at individual timepoints.

Looking at the different microscopic phenomenological dynamic models, we notice that they were developed to simulate a voxel-per-voxel pattern of local ischemia using a simplified 1D or 2D geometry of the brain and a set of empirical parameters [34, 35]. Although these enabled some progress to be made in the microscopic simulation field of modeling spatiotemporal evolution of ischemia, such as tissue heterogeneity and 3D representation of the brain [36], none of the dynamic models were assessed using animal or human data or used information related to visually measurable (or imaging-derived) quantities on medical images (e.g., lesion surface, shape, boundary, intensity etc) [25]. When compared to these approaches, our 4D model presents a more realistic simulation of the dynamics of stroke evolution as it is based on longitudinal MR observations of perfusion and diffusion abnormalities and accounts for the role of time in altering lesion shape and tissue fate.

The model also has some key limitations, particularly when applied to a disease such as stroke: the major limitations are that lesions are commonly not a single defect but commonly consist of multiple scattered smaller abnormalities, that the number of these lesion components may change over time, that stroke lesions swell up and shrink, and that no information derived from tissue parameters such as actual perfusion values can be incorporated directly into the model. For the latter a different modeling approach would be needed.

Despite the small sample of only eight representative patients, we saw wide variance in the spatiotemporal interaction between PWI and DWI lesion surfaces in terms of correspondence between areas of high contraction and expansion (Figures 5 and 6). We also saw significant dynamic changes in MTT lesions, including expansion as well as contraction including in 6 of 8 patients the DWI lesion expanding more rapidly in areas of MTT expansion than in areas where MTT was static. Thus, as well as dynamic contraction and expansion patterns that were in line with that expected from mismatch theory (Figure 5), we saw the DWI lesion surface contracting faster than the corresponding MTT and the hypoperfused MTT surface expanding faster than corresponding DWI. This means that this approach can be used to understand lesion evolution in much greater detail and in relation to anatomic, patient-specific, stroke-specific and treatment-specific, factors than is possible through analysis of 2D or 3D image data. Through analysis of evolution of mean speed of lesion change at different timepoints of DWI and MTT lesions (Figure 7), we identified an overall common pattern implying that diffusion lesions tend to change more slowly than MTT lesions (pink curves and red curves, resp.). We also saw that DWI hyperintense tissue can contract (DWI reversal phenomenon), consistent with recent data [37]. There were variable rates of expansion and contraction of the DWI lesion (blue and pink curves, resp., Figure 7) demonstrating that the DWI lesion surface change is heterogeneous [38].

The model also highlighted wide interpatient variability in the time at which the estimated MTT and DWI evolution scenarios matched the final T2 lesion. In 5 patients, the geometric concordance between the moving acute lesion surface and the static final T2 reached its maximum later in DWI data than in MTT (Table 2). We also saw that the DWI lesion surface did not necessarily converge towards the final T2, although both overlapped partly at some point, again consistent with the theory that DWI abnormal tissue can survive. We also saw that the acute MTT lesion matched the T2 lesion at an earlier stage than for DWI consistent with the hypothesis that failure to reperfuse the perfusion lesion will result in tissue death, at least in some areas. However we also saw in 3 cases that there was little overlap between the static final T2 and the dynamic DWI or MTT lesions (Table 2). Larger studies using this approach and an accurate analysis of the speed of DWI and PWI lesion evolution in a much more diverse range of patients are now justified to determine reasons for these variations in lesion behavior.

Our study has limitations. We were not able to differentiate dynamic lesion behavior in white and grey matter [39], although the blood flow levels and ability to withstand ischemia differ between these tissues. Many of the original 48 patients were excluded at this proof-of-concept stage because their lesions consisted of a changing number of components or they did not have both PWI and DWI lesions visible at all 3 timepoints. However scattered multifocal lesions are common in stroke and would need to be included in future developments [40]. We did not address patient-related factors which might influence lesion evolution such as age, leukoaraiosis, or stroke-specific factors such as timing of vessel recanalisation, as these were beyond the scope of the present development stage. However these are the subject of future work.

Future technical developments should include the ability to model ischemic lesions variation in the number of components, to address anatomical deformation resulting from lesion swelling rather than true lesion expansion, and to use individual voxels throughout the lesion—for example, derived from DWI/PWI values—rather than just the surface/outline. Indeed, investigating the connection between the estimated speed of the surface evolution and corresponding PWI or DWI values would be very interesting, but this requires radical changes in the mathematical formulation of currents to define “colored” surfaces. Coloring the lesion surface mesh with a perfusion or a diffusion value and tracking their change both in intensity and velocity are an ambitious future improvement of the applied model which is beyond the scope of our current study.

Further testing of the model requires larger data sets including more patient-specific variables and stroke-specific variables such as the site of vascular occlusion as well as acute treatment effects.

## 5. Conclusions

To our knowledge this is the first attempt to estimate a continuous 4D PWI and DWI ischemic lesion evolution and use it to evaluate individual patient differences in stroke lesion evolution. Our blinded and prior-free individualized analysis of 8 representative patients of the correspondence in dynamic evolution of DWI and MTT lesions in space and time showed some similarities but also differences in the way the ischemic lesion progressed or resolved. Some of the observations in this proof-of-concept model were partly in line with the mismatch concept, while others contradicted expected lesion behaviors stroke evolution hypotheses such as that acute diffusion abnormality is irreversible and cannot exceed the boundaries of acute perfusion abnormality. We were also able to detect subtle and rapid differences in lesion evolution between DWI and MTT lesions imputed to 3 hourly intervals. The model allows individual lesions and patients to be examined to provide greater insights in speed and time as to what drives stroke lesion evolution and thus other opportunities for developing further interventions. In future this may in turn enhance understanding of stroke pathophysiology and allow greater patient-specific personalized treatment.

## Supplementary Material

These videos show the diffusion and perfusion lesions as they deform in space and time and visualize the different expanding areas in both lesions. Video 1 displays the estimated spatiotemporal evolution scenario of MTT lesion (in red) and DWI lesion (in blue) from acute to subacute timepoints (video 1). Videos 2 and 3 are dynamic translations of Figure 5 (see manuscript): in video 2 you can see the “lighting up” process of successively highly expanding DWI regions along the evolution process, all visualized on the baseline surface at the first acquisition timepoint t1; and in video 3 you can see consecutively highly expanding MTT regions “lighting up” from acute to subacute stages, all mapped back into the baseline MTT lesion surface acquired at timepoint t1.

## Figures and Tables

**Table 1:** Evaluation of the estimated DWI/MTT lesion evolution scenarios. The mean dice index value (a measure quantifying the volumetric overlap between two volumes and ranges from 0 to 1 for the best match) and its standard deviation are computed over the 8 patients between the estimated and the manually delineated DWI/MTT lesions at *t*_2_ and *t*_3_. They are also computed for the additionally spatial deformation *ϕ*_*t*_1__—mapping DWI lesion into MTT lesion at *t*_1_—to show how good is the mapping of DWI lesion into MTT lesion at *t*_1_.

	DWI(*t*_1_)	DWI(*t*_3_)	DWI(*t*_1_) to MTT(*t*_1_)	MTT(*t*_2_)	MTT(*t*_3_)
Mean (dice)	0.90	0.90	0.86	0.76	0.80
SD (dice)	0.02	0.02	0.06	0.09	0.08

**Table 2:** Insights into final T2 outcome Columns 1 and 3 show the mean value of dice index over time for the 8 patients between the estimated evolving DWI/MTT lesion boundaries from acute to subacute stages and the final T2 lesion boundary. Columns 2 and 4 show the time corresponding to the maximum value that the dice index reached, where the final T2 lesion is spatially closest to the estimated DWI/MTT evolution. Columns 5 and 7 display the mean value of the symmetric distance over time computed in mm (the spatial discrepancy) between the evolving DWI/MTT boundaries and the final T2 boundary. Columns 6 and 8 depict the time of the “appearance” (in hours) of the T2 within the DWI and MTT evolution scenarios corresponding to the minimum of the symmetric distance that varied with time—for each patient.

Mean_*t*_ dice_MTT_(*t*)	*t*_MTT,T2_^dice^	Mean_*t*_ dice_DWI_(*t*)	*t*_DWI,T2_^dice^	Mean_*t*_ *d*_sym_ _MTT_(*t*)	*t*_MTT,T2_^*d*_sym_^	Mean_*t*_ *d*_sym_ _DWI_(*t*)	*t*_DWI,T2_^*d*_sym_^
0.574	**30**	0.682	**144**	6.109	**42**	4.719	**144**
0.598	**9**	0.817	**129**	7.086	**9**	3.828	**132**
0.052	**216**	0.029	**216**	33.23	**216**	24.285	**216**
0.003	**3**	0.227	**216**	28.593	**3**	16.681	**216**
0.007	**123**	0.0008	**123**	23.886	**123**	26.128	**120**
0.364	**273**	0.556	**273**	9.295	**270**	6.151	**99**
0.42	**54**	0.61	**6**	10.45	**87**	6.160	**6**
0.681	7**5**	0.77	**198**	7.925	**69**	4.713	**237**

## Appendix

### Regression Criterion

The estimation of the final smooth deformation trajectories between lesion shapes is achieved by minimizing a temporal regression functional *J*(*v*) as follows:

(A.1) $$\begin{array}{c} J\left(v\right)=\sum_{t_{i}}A_{i}\left(x_{1}^{v}\left(t_{i}\right),\ldots ,x_{N}^{v}\left(t_{i}\right)\right)+\gamma \int_{0}^{T}{||v_{t}||}_{V}^{2}dt,\\ v_{t}\left(x\right)=\sum_{p=1}^{N}K^{V}\left(x,x_{i}\left(t\right)\right)\alpha_{i}\left(t\right),\\ x_{i}^{v}\left(t\right)=\chi_{t}^{v}\left(x_{i}\right)=x_{i}+\int_{0}^{t}v_{s}\left(x_{i}\left(s\right)\right)ds,\\ A_{i}={||{\left(\chi_{t_{i}}^{v}\right)}_{*}(S_{0})-S_{i}||}_{W^{*}}^{2}, \end{array}$$

where the fidelity term *A*_*i*_ is a squared similarity measure in the space of currents *W** between the original shape *S*_*i*_ associated with timepoint *t*_*i*_ (in our case the MR observation of the perfusion or diffusion ischemic lesion) and the measured one as a deformation of the baseline surface *S*_0_ at time *t*_*i*_ of the estimated evolution scenario. The action term (*χ*)_∗_(*S*_0_(*x*)) denotes the morphological deformation of each point *x* of the surface *S*_0_ as it moves to the new position *χ*(*x*), and each normal vector *α*(*x*) is deformed into the vector *d*_*x*_ *χ*(*α*), according to the Jacobian matrix of the deformation *d*_*x*_ *χ*. The second energy term denotes the kinetic energy embodying the regularity of the final deformation *χ*_*T*_^*v*^ as a geodesic distance between Id and *χ*_*T*_^*v*^ measured by integrating the norm of the speed vector over time: *d*_*V*_^2^(Id, *χ*_*T*_^*v*^) = ∫_0_^1^||*v*(*t*)^2^||*dt*. The parameter *γ* represents the trade-off between both fidelity to data and regularity terms. This inexact longitudinal matching deformation formulation nicely guarantees the existence and the uniqueness of the solution. In other words, solving the flow equation comes to minimize the kinetic energy the baseline shape needs to *deform itself* onto the following observed lesion shapes, jointly with the least-squared error of “how close” the estimated moving lesion surface gets to the consecutive time-indexed original MR observations of the lesion surface. For more technical details, we refer the reader to Durrleman's Thesis [28].
